# Serial-omics characterization of equine urine

**DOI:** 10.1371/journal.pone.0186258

**Published:** 2017-10-13

**Authors:** Min Yuan, Susanne B. Breitkopf, John M. Asara

**Affiliations:** 1 Division of Signal Transduction, Beth Israel Deaconess Medical Center, Boston, Massachusetts, United States of America; 2 Department of Medicine, Harvard Medical School, Boston, Massachusetts, United States of America; National Research Council of Italy, ITALY

## Abstract

Horse urine is easily collected and contains molecules readily measurable using mass spectrometry that can be used as biomarkers representative of health, disease or drug tampering. This study aimed at analyzing microliter levels of horse urine to purify, identify and quantify proteins, polar metabolites and non-polar lipids. Urine from a healthy 12 year old quarter horse mare on a diet of grass hay and vitamin/mineral supplements with limited pasture access was collected for serial-omics characterization. The urine was treated with methyl tert-butyl ether (MTBE) and methanol to partition into three distinct layers for protein, non-polar lipid and polar metabolite content from a single liquid-liquid extraction and was repeated two times. Each layer was analyzed by high performance liquid chromatography—high resolution tandem mass spectrometry (LC-MS/MS) to obtain protein sequence and relative protein levels as well as identify and quantify small polar metabolites and lipids. The results show 46 urine proteins, many related to normal kidney function, structural and circulatory proteins as well as 474 small polar metabolites but only 10 lipid molecules. Metabolites were mostly related to urea cycle and ammonia recycling as well as amino acid related pathways, plant diet specific molecules, etc. The few lipids represented triglycerides and phospholipids. These data show a complete mass spectrometry based—omics characterization of equine urine from a single 333 μL mid-stream urine aliquot. These omics data help serve as a baseline for healthy mare urine composition and the analyses can be used to monitor disease progression, health status, monitor drug use, etc.

## Introduction

Over the last two decades, mass spectrometry has been used extensively to characterize the protein and small molecule content in biological samples [1]. Mass spectrometry including gas chromatography and liquid chromatography has been used extensively to profile molecules in urine, both from animals and humans [2, 3]. Methods have been developed and evaluated for preparing horse urine samples for small molecule analysis including precipitation and liquid-liquid extraction methods [4]. High resolution mass spectrometry and speed of fragmentation scanning has allowed proteins to be profiled from many diseases tissues and mammalian proteomes including relative protein levels and post-translational modifications of proteins [1, 5–7]. In addition, lipidomics profiling has recently become popular in present day—omics technologies using mass spectrometry (MS) [8]. The tri-ome (proteomics, metabolomics and lipidomics) has been studied by our group from human cell lines in diseases such as cancer as well as mouse tumor tissues [9]. The tri-ome can reveal active pathways that are driving the biology of the organism or tissue in question at the point in time of sampling. Information can be obtained from metabolic pathways, protein synthesis and expression, signal transduction and fatty acid biosynthesis. When studying biofluids such as urine or blood serum, one can also garner information about secreted molecules that are indicative of external influences or internal influences on an organism’s biological state. Equine urine has been tested for several decades in the performance horse industry for the presence of drugs and illegal substances in the racing, showing, eventing and Olympic industries using both GC-MS and LC-MS [10–29]. However, one can also begin to think about profiling the widely accessible fluid for performance markers or measures of health. A recent article utilized a multi-omics proteomic and metabolomic strategy to probe urinary diseases [30]. In this study, we incorporate a “serial-omics” or triomics approach to help create a baseline of normal horse urine in terms of its protein, metabolite and lipid content for the potential diagnosis, treatment and prevention of equine diseases. It uses four different LC-MS/MS platforms including targeted metabolomics, untargeted metabolomics, untargeted lipidomics and untargeted proteomics including phosphoproteomics. The requirement of all platforms is that there is fragmentation to support chemical structure and amino acid sequence with the help of commercial software and publicly available databases for their identification. JF Sierra Flame is an American Quarter Horse Association (AQHA) registered mare foaled in May, 2004. She is fed a daily diet of primarily forage (~20–22 lb) with 2 cups of a fortified grain pellet supplement containing protein, vitamins, minerals, probiotics, etc. in addition to occasional feeding of natural treats such as apples and/or carrots. She is ridden approximately three times per week and has access to a pasture containing grass and some weeds. On rare occasion, if soreness is suspected, the horse is fed Bute-Less pellets (Absorbine) containing the natural anti-inflammatory herbs Devil’s claw and Yucca extract. This study represents molecules that we could accurately detect in horse urine sample using various mass spectrometry approaches that are common to our laboratory and some may be potentially used as biomarkers with rigorous testing.

## Material and methods

### Liquid-liquid extraction

~45 mL of horse mare urine was collected in mid-stream using a 50 mL polypropylene tube. A 333 μL aliquot of horse urine was taken and 2.475 mL HPLC grade methanol (Pharmco-Aaper, #33900HPLC) was added and vortexed vigorously for 1 min. After the addition of 8.25 mL of 99.8% MTBE (Sigma Aldrich, #306975-1L), the samples were shaken for 1 hr at RT. We added 2.06 mL of water, vortexed for 1 min and spun for 10 min. The resulting upper (lipid) and lower (metabolite) liquid phases were collected separately in 1.5 mL glass vials and dried out in a SpeedVac.

### Proteomics

The protein pellet on the bottom was re-suspended in 200 μL 0.5x sample buffer (6X SDS Sample Buffer (0.375M Tris pH 6.8, 12% SDS, 60% glycerol, 0.6M DTT, 0.06% bromophenol blue) transferred to a microcentrifuge tube and dried down to 50 μL in a SpeedVac. The protein samples were loaded on a 4–12% gradient gel (Lonza, #58520) and ran until the loading dye reached the bottom of the gel. The gel was stained with GELCODE Blue (Fisher Scientific, #PI24590) for 30 minutes and each lane with sample was cut into 10 equal pieces. Gel sections were reduced with 55 mM dithiothreitol (DTT) (Sigma-Aldrich), alkylated with 10 mM iodoacetamide (Sigma-Aldrich), and digested overnight with TPCK modified trypsin (Promega) at pH = 8.3. Peptides were extracted, dried out in a SpeedVac, re-suspended in 10 μl of 50% ACN, 6% TFA and rocked on a shaker for 15 min. The TiO_2_ TopTip (PolyLC, # TT10TIO) were washed with 50% ACN, 6% TFA, spin at 1500 rpm 0.5 min for four times. The samples were loaded on the TiO_2_ tips and incubated for 30 min followed by wash with 10 μl 50% ACN, 1% TFA (spin at 1500 rpm 0.5 min), repeated two times, eluted with three times 10 μl 40% ACN, 15% NH_4_OH, added 60 μl buffer A (0.1% formic acid/99.9% water) and dried out to 5 μL. The protein sample was analyzed by positive ion mode LC-MS/MS using LTQ Elite hybrid ion trap-Orbitrap mass spectrometer (Thermo Fisher Scientific) in a data-dependent analysis (DDA Top8). Peptides were delivered and separated using an EASY-nLC nanoflow HPLC (Thermo Fisher Scientific) at 300 nL/min using self-packed 15 cm length × 75 μm i.d. C_18_ fritted microcapillary columns. Solvent gradient conditions were 120 minutes from 3% B buffer to 38% B (B buffer: 100% acetonitrile; A buffer: 0.1% formic acid/99.9% water). MS/MS spectra were analyzed using Mascot search engine v2.5.1 (Matrix Science) by searching the reversed and concatenated Equus caballus (Horse) protein database (UniProt, version 20161102, 20,312 entries) with a parent ion tolerance of 18 ppm and fragment ion tolerance of 0.80 Da. Carbamidomethylation of cysteine (+57.0293 Da) was specified as a fixed modification and oxidation of Methionine (+15.9949), phosphorylation of Serine/Threonine/Tyrosine (+79.97) as variable modifications. Results were imported and analyzed using ScaffoldQ+S 4.6 software (Proteome Software, Inc.) resulting in a peptide false discovery rate (FDR) of ~1%. Further pathway analysis was performed by Panther (http://www.pantherdb.org/).

### Lipidomics

The dried lipid layer was re-suspended in 30 μL of 1:1 LC/MS grade isopropanol:methanol prior to LC-MS/MS analysis, 5 μL were injected. A Cadenza 150 mm x 2 mm 3 μm C_18_ column (Imtakt) heated to 40°C at 260 μL/min was used with a quaternary pump HPLC with room temperature autosampler (Agilent 1100 series). Lipids were eluted over a 20 min. gradient from 32% B buffer (90% IPA/10% ACN/10 mM ammonium formate/0.1% formic acid) to 97% B. A buffer consisted of 59.9% ACN/40% water/10 mM ammonium formate/0.1% formic acid. Lipids were analyzed using a hybrid QExactive Plus Orbitrap mass spectrometer (Thermo Fisher Scientific) in DDA mode using positive/negative ion polarity switching with 1 MS1 scan followed by 8 MS2 HCD scans per cycle (Top 8). DDA data were acquired from m/z 200–1450 in MS1 mode and the resolution was set to 70,000 for MS1 and 35,000 for MS2. MS1 and MS2 target values were set to 5e5 and 1e6, respectively. Lipidomics data were analyzed using LipidSearch 4.1.9 software (Thermo Fisher Scientific) for identification and validation.

### Metabolomics

Half of the metabolite layer was re-suspended in 20 μL LC/MS grade water, 5 μL were injected over a 15 min gradient using a 5500 QTRAP triple quadrupole mass spectrometer (AB/SCIEX) coupled to a Prominence UFLC HPLC system (Shimadzu) via SRM of a total of 287 SRM transitions using positive and negative polarity switching corresponding to 259 unique endogenous water soluble metabolites. Samples were separated using a Amide XBridge HPLC hydrophilic interaction liquid chromatographic (HILIC) column (3.5 μm; 4.6 mm inner diameter (i.d.) × 100 mm length; Waters) at 300 μL/min. Gradients were run starting from 85% buffer B (LC/MS grade acetonitrile) to 40% B from 0–5 min; 40% B to 0% B from 5–16 min; 0% B was held from 16–24 min; 0% B to 85% B from 24–25 min; 85% B was held for 7 min to re-equilibrate the column. Buffer A was comprised of 20 mM ammonium hydroxide/20 mM ammonium acetate (pH = 9.0) in 95:5 water/acetonitrile. Peak areas from the total ion current for each metabolite SRM transition were integrated using MultiQuant version 2.1.1 software (AB/SCIEX). The other half of the metabolite layer was re-suspended in 20 μL LC/MS grade water, 5 μL were analyzed by positive/negative polarity switching mode using a hybrid QExactive HF Orbitrap mass spectrometer (Thermo Fisher Scientific) via a data-dependent analysis (DDA Top8). Metabolites were delivered and separated using an EASY-nLC nanoflow HPLC (Thermo Fisher Scientific) at 225 nL/min using self-packed 15 cm length × 75 μm i.d. C_18_ fritted microcapillary columns. Solvent gradient conditions were 25 minutes from 3% B buffer to 38% B (B buffer: 100% acetonitrile; A buffer: 0.1% formic acid/99.9% water). The data were analyzed using Elements (Proteome Software) with the NIST MS/MS spectral database (http://chemdata.nist.gov/mass-spc/msms-search/) and HMDB metabolite database followed by statistical analysis and pathway analysis with Excel 2013 and MetaboAnalyst 3.0 software (http://www.metaboanalyst.ca/).

## Results and discussion

**Fig 1:**
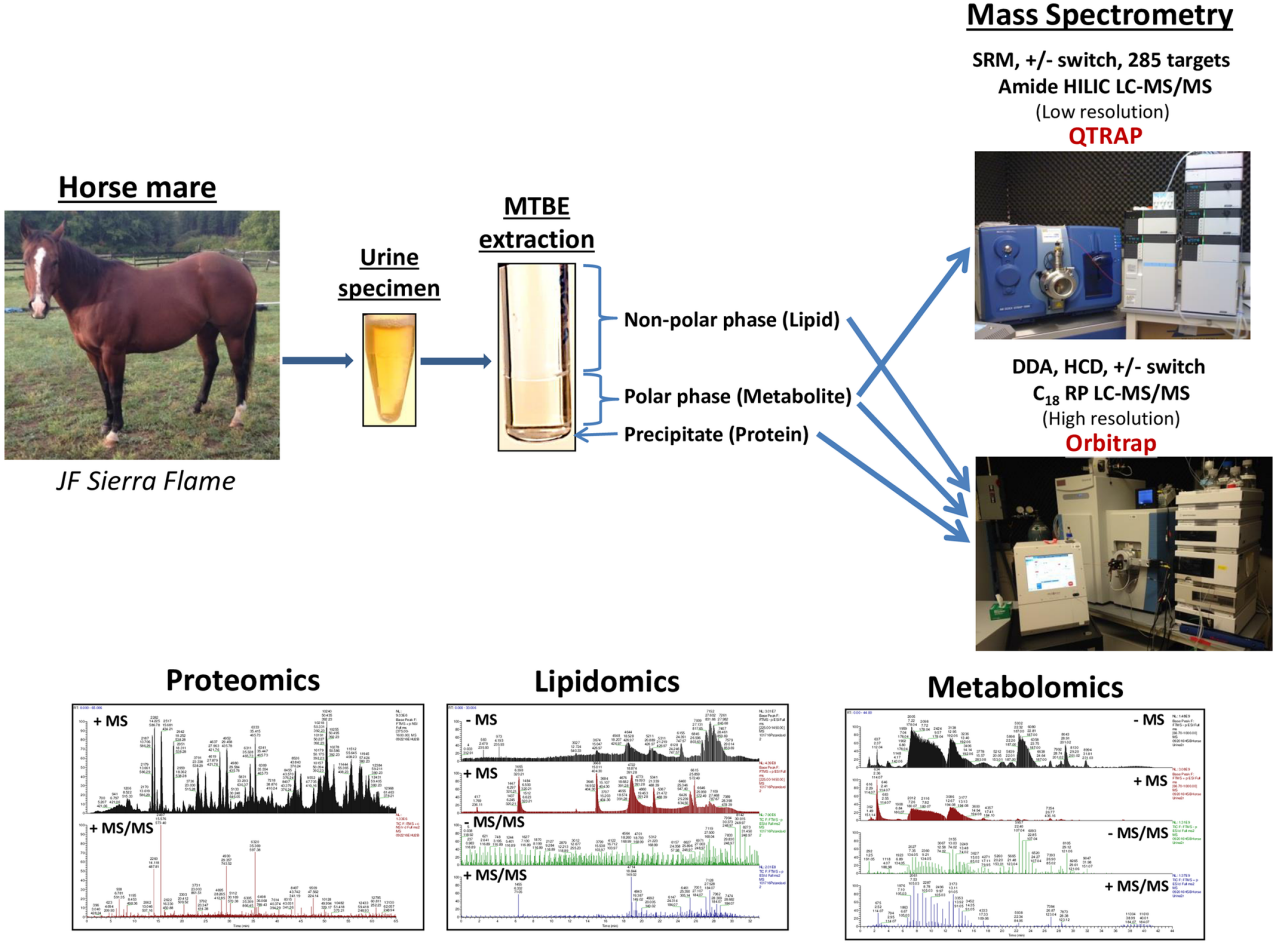
The workflow of the serial-omics analysis of horse urine from collection and MTBE extraction through high resolution tandem mass spectrometry (LC-MS/MS) analysis. Each phase of the MTBE extraction is used for either proteomics, metabolomics or lipidomics analyses using untargeted or targeted mass spectrometry in order to gather the—omics profile.

We utilized a new approach whereby a single aliquot of urine is used for each individual—omics analysis via a liquid-liquid extraction with methyl tert-butyl ether (MTBE). When MTBE, methanol and water are added to urine, the protein precipitates to the bottom while the aqueous polar metabolites form a middle layer and the non-polar lipids form the top layer. Each layer including the protein precipitate was removed and analyzed separately. Fig 1 shows the workflow from the horse anatomy to urine collection, sample preparation, mass spectrometry analysis and the data analysis. The protein mixture was separated into 10 fractions using SDS-PAGE gel [31] and in gel digested using trypsin to produce sequenceable peptides. Each peptide mixture was analyzed using a 1hr LC-MS/MS data dependent acquisition (DDA) run with nanoflow HPLC and a hybrid Orbitrap Elite high resolution mass spectrometer in biological triplicates.

The results after database searching and interpretation are shown in Table 1. We identified 46 unique proteins and they are ranked according to the number of total peptide spectra identified (spectral count) which can be indicative of relative protein quantity.

**Table 1:** Equine mare urine proteins identified by LC-MS/MS from a serial-omics liquid-liquid extraction.

Protein name	Accession	MW	# of spectra
Uromodulin OS = Equus caballus	F7BM54_HORSE	70 kDa	376
Calcium-activated chloride channel regulator 1 OS = Equus caballus	CLCA1_HORSE	100 kDa	101
Keratin, type I cytoskeletal 10 OS = Equus caballus	F6WDW3_HORSE	43 kDa	66
Protein AMBP OS = Equus caballus	F6UZH0_HORSE	39 kDa	56
Immmunoglobulin lambda light chain OS = Equus caballus	F6QAU5_HORSE	11 kDa	47
Keratin, type II cytoskeletal 1 OS = Equus caballus	F7B7X0_HORSE	66 kDa	44
Kininogen-1 OS = Equus caballus	F7C0Z0_HORSE	72 kDa	42
Keratin, type II cytoskeletal 5 OS = Equus caballus	F6W7V0_HORSE	62 kDa	33
Cadherin 1 OS = Equus caballus	F6Y0D9_HORSE	89 kDa	26
Poly-Ig receptor OS = Equus caballus	F6W2K5_HORSE	83 kDa	26
EGF-containing fibulin-like extracellular matrix protein 1 OS = Equus caballus	F6PVG3_HORSE	55 kDa	25
Cytokeratin-2e OS = Equus caballus	F6SHJ8_HORSE	61 kDa	24
Pro-epidermal growth factor OS = Equus caballus	F7B762_HORSE	133 kDa	23
Keratin, type II cytoskeletal 6A OS = Equus caballus	F7AGY4_HORSE	60 kDa	21
Keratin, type I cytoskeletal 16 OS = Equus caballus	F6ZEQ3_HORSE	51 kDa	20
Ubiquitin A-52 residue ribosomal protein fusion product 1 OS = Equus caballus	A0A0B4J1C5_HORSE	15 kDa	19
Pantetheinase OS = Equus caballus	F6Z129_HORSE	58 kDa	17
Antithrombin protein OS = Equus caballus	F7CYR1_HORSE	52 kDa	17
Trypsinogen OS = Equus caballus	F6VNT7_HORSE	26 kDa	16
Epithelial cell adhesion molecule OS = Equus caballus	F6R6Z6_HORSE	33 kDa	16
Keratin, type II cytoskeletal 73 OS = Equus caballus	F7C7Y1_HORSE	59 kDa	16
Cadherin-6 OS = Equus caballus	F6X200_HORSE	88 kDa	14
Corticosteroid-binding globulin OS = Equus caballus	F7DRS2_HORSE	45 kDa	14
Complement C4-A OS = Equus caballus	F6XSF7_HORSE	193 kDa	13
Heparan sulfate proteoglycan 2 OS = Equus caballus	F7C0I7_HORSE	466 kDa	12
Butyrophilin-like protein 10 OS = Equus caballus	F6VGK4_HORSE	51 kDa	12
Collagen alpha-3(VI) chain OS = Equus caballus	F6R735_HORSE	342 kDa	11
IgG heavy chain OS = Equus caballus	H9GZT5_HORSE	36 kDa	11
Cadherin-15 OS = Equus caballus	F6WEI6_HORSE	82 kDa	11
Tenascin-X OS = Equus caballus	F7CCQ6_HORSE	445 kDa	9
Desmocollin-2 OS = Equus caballus	F6UVP2_HORSE	100 kDa	8
Keratin, type I cytoskeletal 17 OS = Equus caballus	F6YIA9_HORSE	48 kDa	8
Mucin-5AC OS = Equus caballus	F6QC83_HORSE	477 kDa	7
Aminopeptidase N OS = Equus caballus	F7B847_HORSE	110 kDa	6
Major allergen Equ c 1 OS = Equus caballus	ALL1_HORSE	22 kDa	6
Cartilage intermediate layer protein OS = Equus caballus	F7C2J3_HORSE	133 kDa	5
Tetratricopeptide repeat protein 28 OS = Equus caballus	F6WBY4_HORSE	257 kDa	5
Protein FAM151A OS = Equus caballus	F6WIV4_HORSE	64 kDa	4
Serum albumin OS = Equus caballus	ALBU_HORSE	69 kDa	4
Microtubule-actin cross-linking factor 1 OS = Equus caballus	F6YMD9_HORSE	827 kDa	3
Collagen alpha-1 type I chain OS = Equus caballus	F7A3F7_HORSE	141 kDa	3
Nuclear receptor co-repressor 2 OS = Equus caballus	F6UWM2_HORSE	275 kDa	3
Synapsin-1 isoform Ib OS = Equus caballus	F6XVE9_HORSE	58 kDa	3
AXL receptor tyrosine kinase OS = Equus caballus	F6VEV4_HORSE	98 kDa	3
Nuclear receptor-interacting protein 1 OS = Equus caballus	F6QWB9_HORSE	127 kDa	3
Far upstream element-binding protein 2 OS = Equus caballus	F7A984_HORSE	70 kDa	3

**Fig 2:**
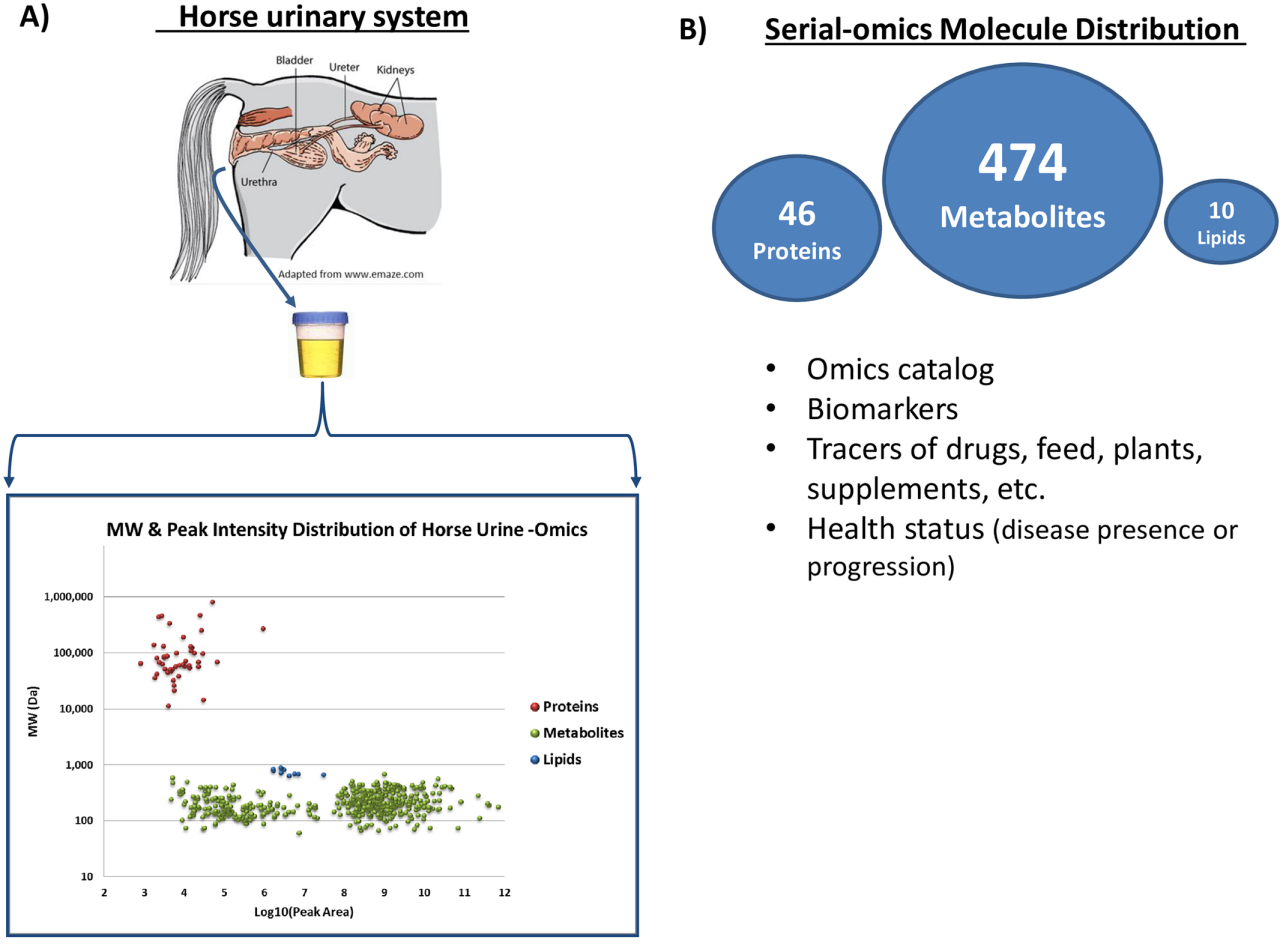
**A)** Omics results from the urinary tract and specimen cup to the number of unique molecules identified from horse urine. A scatterplot showing the molecular weight distribution of lipids, metabolites and proteins. Reprinted from The Merck Manual for Pet Health under a CC BY license, with permission from Merck & Co., original copyright 2017. **B)** The ratio of metabolite:protein:lipid was approximately 10.0:1.0:0.22. The majority of urine content is small polar molecules (474) followed by a significant amount of proteins (46) and very low number of lipids (10). A short list of some example questions that can be addressed using a serial-omics approach from accessible bodily fluids such as urine, blood, etc.

Proteomics is very common for detecting biomarkers, quantifying protein levels across sample conditions and for identifying proteins and post-translational modifications (PTMs) [6, 32, 33] including urinary proteomics [34]. Over 500 proteins were reported from canine urine [35], though other articles report far less proteins in mammalian urine [36]. For example, one report showed less than 100 proteins in human urine [37]. The urinary proteome varies greatly from animal to animal or patient to patient, etc. due to many factors including health, diet, medications, lifestyle, etc. and there is no benchmark of expected numbers. We identified a total of 46 unique proteins from 12 yr old quarter horse mare urine (Fig 2A and 2B). We required at least 2 unique peptides per protein and a false discovery rate (FDR) of less than 1% for a positive identification from the Uniprot Equus caballus (horse) protein database. The top five proteins other than keratin, a common cytoskeletal protein related to skin and hair, are common to the urinary tract and kidneys and include uromodulin, calcium activated chloride channel protein, protein AMBP, kininogen-1, the cell adhesion cadherin molecules 1, 6, 15 and epithelial cell adhesion molecule, etc. In addition, structural and cytoskeletal proteins were identified as well as other glycoproteins commonly expressed in connective tissue such as tenascin-X. Pantetheinase is secreted from the mucous membranes of the intestine to hydrolyze pantotheine to pantothenate (Vitamin B) ([38, 39]. Butyrophilin-like protein 10 is an intestinal epithelial protein containing an Ig domain and may regulate T lymphocytes [40, 41]. Trypsinogen is a precursor of the enzyme trypsin and is produced in the pancreas and its levels are sometimes used as marker for pancreatitis [42]. The corticosteroid-binding globulin protein binds circulating plasma cortisol and may play hormonal roles, possibly in mares, especially during pregnancy when its plasma concentration decreases [43, 44]. The bottom portion of Fig 2A shows a scatterplot that demonstrates the molecular weight distribution vs relative intensity between lipids, polar metabolites and proteins identified across all LC-MS/MS based—omics experiments from the urine aliquot. These data show that the majority of urinary components are small molecules followed by very large protein molecules and far fewer moderately sized lipids. A detailed description of the Fig 2B shows a simple number distribution of the various omics results and the bottom portion of Fig 2B lists a number of topics that we can address using serial-omics. For example, a single aliquot of microliter levels of urine, blood, etc. can be used collect a catalog of molecules from small polar metabolites to non-polar lipids to large proteins and the collection of these molecules may be used to develop biomarkers for tracers of drugs, feed, plants, supplements, etc. in addition to monitoring health or disease status and markers of performance and recovery. However, defining and validating biomarkers of this sort requires the collection and analysis of urine from a large number (>50) of healthy horses of varying age and gender in order to generate a baseline of “normal” serial-omics profiles [45]. However, using targeted mass spectrometry for the quantification of potential biomarkers is a routine and robust approach [46].

**Fig 3:**
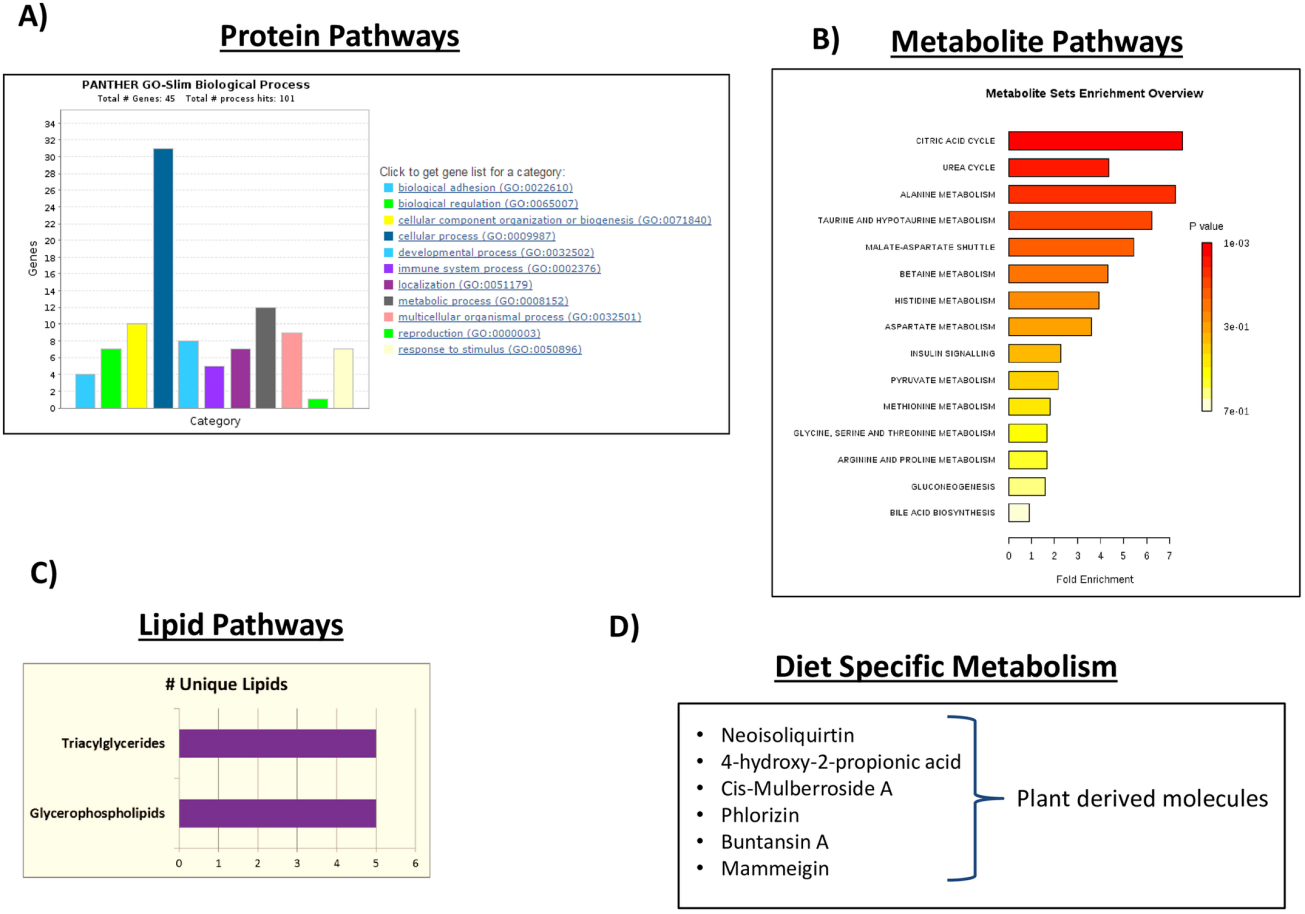
Biological pathways represented by serial-omics of healthy horse mare urine. **A)** Panther biological processes from proteomics data analysis. **B)** MetaboAnalyst metabolite enrichemnt sets from polar metabolomics data. **C)** The few identified lipids were represented by triglycerides and glycerophospholipids. **D)** Plant metabolites identified as horse diet specific.

Fig 3A shows the enriched representative protein pathways using the Panther gene ontology tool [47]. Many other proteins were detected in the normal mare urine and are listed in the S1 Dataset containing the peptide sequences identified as well as the database search criteria and scoring information.

After characterizing the urine proteome of the 12 year old quarter horse mare, we analyzed the aqueous polar metabolite composition of the MTBE extraction middle layer. We used two different approaches to identify small polar metabolites, a targeted method that utilized positive/negative polarity switching via selected reaction monitoring (SRM) [48] and an untargeted method using a high resolution QExactive HF mass spectrometer in positive/negative polarity switching mode via DDA using Elements identification software and the NIST small molecule spectral database. From 474 total unique metabolites identified from both experiments in triplicate, the major identifiable pathways are attributable to protein biosynthesis due to an abundance of amino acids likely broken down from hay and grain products. In addition, amino acid related pathways such as methionine, alanine, aspartate, glycine, serine, threonine, arginine and proline metabolism amongst several others are well represented in the metabolome of normal urine. These amino acids are breakdown products from digested proteins in hay and grain as well as amino acids that may be present in feed supplements and not utilized by the horse. In addition, prominent urine and kidney related pathways are well-represented including the urea cycle and ammonia recycling and many other major metabolite pathways such as glutathione metabolism, betaine metabolism, fatty acid oxidation, NAD metabolism, etc.

We then took the top 10 metabolites identified from each experiment (targeted and untargeted) for a total of 20 metabolites and performed a pathway enrichment analysis using MetaboAnalyst software (Fig 3B) [49]. The analysis revealed the major pathways are citric acid cycle, urea cycle, betaine metabolism, taurine and hypotaurine metabolism followed by amino acid metabolism. To better resolve the metabolite analysis, we identified the top 3 most intense metabolites from each metabolomics dataset and characterized them. From the untargeted experiment, hippuric acid, a common carboxylic acid found in the urine of horses and other plant eating species was the most abundant molecule identified followed by creatinine, a breakdown product of creatine that is commonly secreted through urine and is used to monitor normal kidney function and can be used as a urine control metabolite for normalization [50]. Phenylacetylglycine was also very abundant and is a gut microbial co-metabolite with hippuric acid and well-studied using LC-MS/MS methods from urine as phospholipidosis markers [51, 52]. p-Cresol glucuronide is another common urine metabolite derived from intestinal bacteria. The targeted SRM based mass spectrometry experiment which is limited to less than 300 metabolites, shows the top molecules as creatinine, betaine, urea, taurine, citrate, etc.; metabolites which are known to be present in high concentrations in urine [4, 53]. The same strategy of taking the top 20 metabolites in MS1 peak intensity from the non-targeted experiment did not yield any known enriched metabolic pathways. The targeted metabolite method can be used to target specific drugs for quantification if one is testing for a particular compound or several compounds [54–57]. Our lab routinely performs that test from mouse tumor tissue and there are many reports in urinalysis [58]. To name a few more abundant urinary horse metabolites, DOPA sulfate levels in urine have been used to assess central nervous system disorders [59] and butyrlcarnitine has been associated with energy metabolism and studied in urine by mass spectrometry for various diseases [60]. Although no drugs were administered in the horse, some compounds used mostly as drugs isolated from natural products such as plants were identified. One example is pilocarpine, a glaucoma drug [61, 62]. Since the horse has a plant based diet, it is not unreasonable that some of these compounds may be consumed during pasture grazing. Table 2 shows the top 60 metabolites by peak intensity (Top 30 by both targeted and untargeted methods) and the S2 Dataset lists all 474 unique identified polar metabolites from both experiments (targeted and untargeted) including peak area quantification and scoring information.

**Table 2:** Top 60 equine mare urine metabolites by intensity from 474 total identified metabolites by LC-MS/MS from a serial-omics liquid-liquid extraction.

**Polar metabolite name (Untargeted, Top 30)**	**Accession**	**Molecular Formula**	**MS1 Log10 Peak Area**
Hippuric acid	HMDB00714	C9H9NO3	11.8
Phenylacetylglycine	HMDB00821	C10H11NO3	11.6
2-Hydroxy-4-trifluoromethyl benzoic acid	HMDB60715	C8H5F3O3	11.6
Creatinine	HMDB00562	C4H7N3O	11.4
p-Cresol glucuronide	HMDB11686	C13H16O7	11.3
Buntansin A	HMDB35086	C11H 8O5	10.9
Acetohydroxamic Acid	HMDB14691	C2H5NO2	10.8
Mesoridazine	HMDB15068	C21H26N2OS2	10.7
Mammeigin	HMDB30785	C25H24O5	10.6
Monomethyl phenylphosphonate	HMDB31868	C7H9O3P	10.6
Neoisoliquiritin	HMDB37317	C21H22O9	10.5
Geranylgeranylcysteine	HMDB11678	C23H37NO3S	10.4
4-Hydroxy-8-methoxy-2H-furo[2,3-h]-1-benzopyran-2-one	HMDB32659	C12H8O5	10.4
N'-Hydroxysaxitoxin	HMDB33664	C10H17N7O5	10.4
4-Hydroxyphenyl-2-propionic acid	HMDB41683	C9H10O3	10.4
cis-Mulberroside A	HMDB31726	C26H32O14	10.3
N-Methylphthalimide	CASNO:550-44-7	C9H7NO2	10.3
Phlorizin	HMDB36634	C21H24O10	10.3
L-leucyl-L-proline	HMDB11175	C11H20N2O3	10.2
Benzocaine	HMDB04992	C9H11NO2	10.2
Vanilloloside	HMDB32013	C14H20O8	10.2
apo-[3-methylcrotonoyl-CoA:carbon-dioxide ligase (ADP-forming)]	HMDB59607	C7H15N3O2	10.1
Trimethylamine N-oxide	HMDB00925	C3H9NO	10.1
Acetaminophen	HMDB01859	C8H9NO2	10.1
D-(-)-Isoascorbic acid	CASNO:89-65-6	C6H8O6	10.1
Butyrylcarnitine	HMDB02013	C11H21NO4	10.1
DOPA sulfate	HMDB02028	C9H11NO7S	10.0
Pilocarpine	HMDB15217	C11H16N2O2	10.0
Pyrogallol-2-O-glucuronide	HMDB60017	C12H14O9	10.0
**Polar metabolite name (Targeted, Top 30)**	**Accession**	**Molecular Formula**	**Q3 Log10 Peak Area**
2-Hydroxy-2-methylbutanedioic acid	C02612	C5H8O5	7.7
citrate	C00158	C6H8O7	7.3
2-Isopropylmalic acid	C02504	C7H12O5	7.3
1-Methyl-Histidine	C01152	C7H11N3O2	7.3
betaine	C00719	C5H11NO2	7.2
aconitate	C00417	C6H6O6	7.1
oxaloacetate	C00036	C4H4O5	7.1
Acetylcarnitine DL	C02571	C9H18NO4	7.1
allantoin	C01551	C4H6N4O3	7.1
Urea	C00086	CH4N2O	6.9
Acetyllysine	C02727	C8H16N2O3	6.8
N6-Acetyl-L-lysine	C02727	C8H16N2O3	6.7
2-hydroxygluterate	C02630	C5H8O5	6.7
D-sedoheptulose-1-7-phosphate	C05382	C7H15O10P	6.6
Phenylpropiolic acid	HMDB00563	C9H6O2	6.6
DL-Pipecolic acid	C00408	C6H11NO2	6.5
Citraconic acid	C02226	C5H6O4	6.5
Ascorbic acid	C00072	C6H8O6	6.5
3-hydroxybuteric acid	C01089	C4H8O3	6.5
Ng,NG-dimethyl-L-arginine	C03626	C8H18N4O2	6.3
Kynurenic acid	C01717	C10H7NO3	6.3
p-hydroxybenzoate	C00156	C7H6O3	6.3
taurine	C00245	C2H7NO3S	6.2
succinate	C00042	C4H6O4	6.2
Aminoadipic acid	C00956	C6H11NO4	6.2
Atrolactic acid	HMDB00475	C9H10O3	6.2
pantothenate	C00864	C9H17NO5	6.1
2-dehydro-D-gluconate	C00629	C6H10O7	6.1
orotate	C00295	C5H4N2O4	6.1
glutamine	C00064	C5H10N2O3	6.1

In addition to the most abundant metabolites in horse urine, some interesting metabolites were identified that are indicative of the feeding habits of both humans and horses (Fig 3D). One metabolite, phlorizin, is a common flavonoid metabolite in high concentration in apples, a common treat fed to horses as well as being present in other vegetative plants, leaves, bark, etc. [63]. Mammeigin is another common fruit and vegetable specific neoflavinoid compound and may have antiproliferative activity in some cancer cells [64]. Buntansin A is a coumerin compound commonly found in plants [65]. A surprising finding is that the second most abundant metabolites from the non-targeted metabolomics search was acetohydroxamic acid (AHA), a urea-like molecule that is synthetically made to treat urinary tract infections, prevent kidney stones and studied in human as well as canine urine [66]. Since we did not administer this drug to the horse, this may be evidence that AHA is produced naturally in the horse and present in the kidneys and subsequently secreted in the urine. A high level of Monomethyl phenylphosphonate was found to be present and this compound is a breakdown product of leptophos, a no longer used pesticide. It is important to note that Bronco Gold fly spray (Farnum) is routinely used on the horse during summer months during fly season. The main active ingredients in the fly spray are pyrethrins and pyrethroids, piperonyl butoxide and butoxy poly propylene glycol. Many other abundant natural metabolites that do not fit into distinct pathways may be derived from either the feed byproducts or plant derivatives and include N'-Hydroxysaxitoxin, 4-hydroxyphenyl-2-propionic acid, cis-mulberroside A, neoisoquirtin, etc.

The last—omics component that we analyzed was lipidomics or any identifiable fatty acid containing molecules in the horse mare urine. Urine typically does not contain an appreciable amount of lipid unless nephrotic syndromes are present in either humans or animals [67]. A study in human prostate cancer yielded only approximately 100 lipid molecules using LC-MS/MS [68]. In general, non-polar lipids should be barely detectable in healthy mammals. We used a QExactive Plus Orbitrap mass spectrometer in polarity switching mode with reversed-phase chromatography with LipidSearch identification software [69, 70]. The results in Table 3 and Fig 3C show that very few lipids were identified. We identified five triglyceride (TG) lipids, two phosphatidic acid (PA) lipids, two phosphatidylethanolamines (PE) and two phosphatidylethanol (PEt) lipids. To put that in perspective, we routinely identify ~1000 lipids or more from cells, plasma, tumors, etc, with our lipidomics platform [8]. The fatty acid chains associated with the identified lipid molecules primarily consisted of the basic fatty acid building blocks of palmitate (C16:0), oleate (C18:1) and stearate (C18:0). The very few identified lipids in horse urine are consistent with expectations for a healthy mammal. The S3 Dataset contains the detailed lipid search results including scoring information and peak area quantification.

**Table 3:** Equine mare urine non-polar lipids identified by LC-MS/MS from a serial-omics liquid-liquid extraction.

Lipid name	Accession	Ion Formula	MS1 Log10 Peak Area
PA(16:0/18:1)-H	LMGP10010032	C37H70O8N0P1	7.473
PA(18:0/18:1)-H	LMGP10010037	C39H74O8N0P1	6.740
PE(16:0/16:0)+H	LMGP02010037	C37H75O8N1P1	6.842
PE(18:0/16:0)+H	LMGP02011205	C39H79O8N1P1	6.407
PEt(16:0/14:0)-H	N/A	C35H68O8N0P1	6.613
TG(16:0/14:0/16:0)+NH4	LMGL03012786	C49H98O6N1	6.224
TG(16:0/16:0/16:0)+NH4	LMGL03010001	C51H102O6N1	6.480
TG(18:0/16:0/16:0)+NH4	LMGL03010004	C53H106O6N1	6.213
TG(16:0/18:1/18:1)+NH4	LMGL03010100	C55H106O6N1	6.415
TG(18:1/18:1/18:1)+NH4	LMGL03012612	C57H108O6N1	6.402

## Conclusions

It is important to note that the majority of global urine -omics studies have taken place from human and mouse urine samples while most horse urine studies have focused on specific targeted compounds. However, we expect that many mammals should have a somewhat similar urine profile as far as it concerns the major metabolites and proteins. However, diet also plays a crucial role as well as differences in the digestion system between horses and humans. As a result, we found a significant number of plant related metabolites due to a horse’s diet of hay and vegetation. As expected, the number of identified proteins (46) from horse mare urine was significant but far less than analyses in other biological tissues such as cells, plasma, solid tissue where thousands of proteins are the norm. The number of small molecule polar metabolites was high with 474 molecules identified. Only 10 non-polar lipid molecules were identified. These show that a single aliquot of horse urine can be used for a liquid-liquid extraction for a multi-omics analysis. We chose to perform lipidomics, proteomics and metabolomics on normal 12 year old horse mare urine. These analyses demonstrate a baseline for omics analyses from horse urine and can be used as a reference for expected future results. This technique can be applied in discovering the presence of a diseased or drug administered-horse from the norm by comparing alterations in the metabolites, lipids and proteins identified. If repeated over a time-course, these analyses can potentially be used to monitor disease progression, health status, inflammation or used to develop biomarkers for performance indicators. Not only do we expect levels of common metabolites and proteins to vary across sample conditions, but unique molecules to be identified in specific cohorts. The proteomic analysis can reveal disease-specific proteins or reveal a relative quantitative change in protein levels. The untargeted metabolomic analyses can identify illicit drug molecules or reveal metabolic changes that indicate disease. In addition, the lipidomics analysis can reveal diseases in the nephrotic system based on the number and types of lipids present. While horses urinate in several liter volumes per episode, we chose to use a very small aliquot of 333 μL in order to demonstrate that we can acquire comprehensive—omics data on small volumes of biological fluid. This can be applied to as little as a few drops of blood, saliva, tears, etc [71]. The current state of high resolution tandem mass spectrometry is extraordinarily sensitive down to sub nanogram levels [72]. These data represent the first comprehensive multi-omics report from normal and healthy horse urine and is intended to be used as a reference tri-ome for further comprehensive equine urine research. We anticipate follow-up studies will include various equine age groups, sexes, in addition to various time points related to pre and post riding exercises from events such as show jumping, dressage, racing, rodeo, polo, etc.

## Supporting information

S1 Dataset Proteomics dataset containing all of the identified horse urine proteins from LC-MS/MS analysis and Mascot database searching. (XLSX)

S2 Dataset Complete polar horse urine metabolomics dataset from both targeted and untargeted LC-MS/MS with polarity switching from Elements database searching. (XLSX)

S3 Dataset Non-polar horse urine lipidomics dataset from untargeted LC-MS/MS with polarity swiching and LipidSearch database search results. (XLS)
